# Macrophage metabolic reprogramming in organ transplantation: mechanisms, transplant outcomes, and therapeutic implications

**DOI:** 10.3389/fcimb.2026.1868319

**Published:** 2026-08-10

**Authors:** Shaofeng Chen, Dejun Kong, Yeqi Nian, Zhenglu Wang, Hong Zheng

**Affiliations:** 1 Nankai University School of Medicine, Tianjin, China; 2 Tianjin First Central Hospital, Organ Transplantation Research Center, Tianjin, China; 3 Liver Transplantation Center, National Clinical Research Center for Digestive Diseases, Beijing Friendship Hospital, Capital Medical University, Beijing, China; 4 Laboratory for Clinical Medicine, Capital Medical University, Beijing, China; 5 Capital Medical University, State Key Laboratory of Digestive Health, Beijing, China; 6 Beijing Key Laboratory of Organ Cultivation and Organ Protection in Transplantation, Beijing Friendship Hospital, Capital Medical University, Beijing, China; 7 Clinical Center for Pediatric Liver Transplantation, Capital Medical University, Beijing, China; 8 Department of Renal Transplantation, Tianjin First Central Hospital, Nankai University, Tianjin, China; 9 Institute of Transplantation Medicine, Nankai University, Tianjin, China; 10 Biological Sample Resource Sharing Center, Tianjin First Central Hospital, Tianjin, China; 11 Tianjin Key Laboratory of Organ Transplantation, Tianjin First Central Hospital, Tianjin, China; 12 Key Laboratory of Transplant Medicine, Chinese Academy of Medical Science, Tianjin, China

**Keywords:** allograft rejection, immunometabolism, ischemia-reperfusion injury, macrophage, metabolic reprogramming, organ transplantation

## Abstract

Organ transplantation is the definitive treatment for end-stage organ failure, yet long-term graft survival remains substantially limited by ischemia-reperfusion injury (IRI), allograft rejection, and chronic graft dysfunction. Current immunosuppressive regimens have not fully exploited the metabolic plasticity of macrophages, which are central orchestrators of both innate and adaptive immune responses in transplanted organs. Macrophages display remarkable functional plasticity, classically defined by pro-inflammatory (M1)/anti-inflammatory (M2) polarization, and this dual capacity renders them uniquely impactful in transplanted organs. Accumulating immunometabolic evidence indicates that this plasticity is governed by dynamic metabolic reprogramming orchestrated by key metabolic nodes: M1 macrophages rely primarily on aerobic glycolysis and secrete proinflammatory cytokines, such as IL-1β, IL-6, and TNF-α, whereas M2 macrophages depend on oxidative phosphorylation (OXPHOS) and fatty acid oxidation (FAO) to sustain anti-inflammatory and tissue-repair programs. For example, pyruvate kinase M2, a key glycolytic enzyme, promotes M1 polarization via glycolytic reprogramming and HIF-1α-dependent inflammatory gene transcription, whereas the carnitine palmitoyltransferase 1A, a rate-limiting enzyme in FAO, supports M2 polarization through FAO-driven OXPHOS. Core metabolic pathways encompass carbohydrate metabolism-glycolysis, the tricarboxylic acid cycle, and the pentose phosphate pathway-alongside FAO and amino acid catabolism. These pathways are dynamically modulated by microenvironmental cues, such as hypoxia, lactate, and succinate, and in turn dictate macrophage phenotypic identity and effector function. In this review, we comprehensively review the molecular mechanisms underpinning macrophage metabolic reprogramming, from early IRI and acute rejection to chronic rejection, fibrosis, and post-transplant tumor recurrence, linking metabolism to alloimmunity and oncological risk. Emerging therapeutic strategies target macrophage metabolism, including metabolic enzyme inhibitors such as 2-deoxyglucose, which attenuates chronic lung allograft dysfunction, cell-based therapies, nanoparticles, and gene editing, for example macrophage-specific MEK1/2 ablation via CRISPR/Cas9, which reprograms glycolysis to OXPHOS and ameliorates cardiac rejection. Harnessing these metabolic nodes may complement current immunosuppression and improve graft survival, warranting future clinical evaluation.

## 1 Introduction

Organ transplantation remains the most effective intervention for patients with end-stage organ failure, however, improving long-term graft survival remains a major clinical challenge. Although adaptive immune responses, particularly T cell-mediated allograft rejection, have long been the primary focus of transplantation immunology, innate immune cells account for approximately 38-60% of allograft-infiltrating leukocytes and exert a profound, yet often underappreciated, influence on graft outcomes (LaRosa et al., 2007; Murphy et al., 2011). Among these innate immune cells, macrophages play a pivotal role in regulating post-transplant immune responses. Macrophages orchestrate the balance between ischemia-reperfusion injury (IRI) and immune tolerance by dynamically shifting between pro-inflammatory and anti-inflammatory phenotypes. This dynamic phenotypic plasticity is a key determinant of both short- and long-term graft outcomes (Kopecky et al., 2022; Lackner et al., 2023; Shen et al., 2024). Therefore, targeting macrophages has emerged as a promising strategy for improving long-term graft survival.

Immunometabolism is an emerging interdisciplinary field that examines how cellular metabolic processes regulate the development, maintenance, and functional states of immune cells, particularly in the context of transplantation. In response to microenvironmental cues, immune cells undergo dynamic metabolic remodeling, in turn, reciprocally shape their cellular functions. Environmental signals, including hypoxia and nutrient deprivation, regulate key metabolic pathways-such as glycolysis and fatty acid oxidation (FAO)-primarily through hypoxia-inducible factor-1α (HIF-1α) and mammalian target of rapamycin (mTOR) signaling. These pathways, in turn, direct macrophage polarization (El Kasmi and Stenmark, 2015).

Targeting macrophage metabolism offers a promising complementary or alternative approach to overcome several limitations of current immunosuppressive agents, including their lack of cellular specificity and associated toxicities. Conventional agents such as calcineurin inhibitors and mTOR inhibitors broadly suppress T cell activation and proliferation but often fail to adequately control macrophage-driven inflammation. For example, infiltrating M1 macrophages produce interleukin-1β (IL-1β) and tumor necrosis factor-α (TNF-α), which exacerbate ischemia-reperfusion injury and acute allograft rejection (Mistry et al., 2025; Nguyen et al., 2019; Nimbarte et al., 2025). In contrast, metabolic interventions offer unique advantages by selectively targeting specific macrophage activation states while preserving the function of regulatory subsets. For instance, glycolytic inhibition can preferentially suppress pro-inflammatory M1 macrophages while preserving oxidative metabolism in regulatory subsets. Furthermore, because metabolic state programs are highly dynamic and can be modulated by microenvironmental cues or pharmacological agents, metabolic reprogramming offers the potential for more precise and better-tolerated therapeutic interventions (Bettencourt and Powell, 2017; Owen and Kopecky, 2024; Viola et al., 2019; Wang et al., 2025).

**Figure 1:**
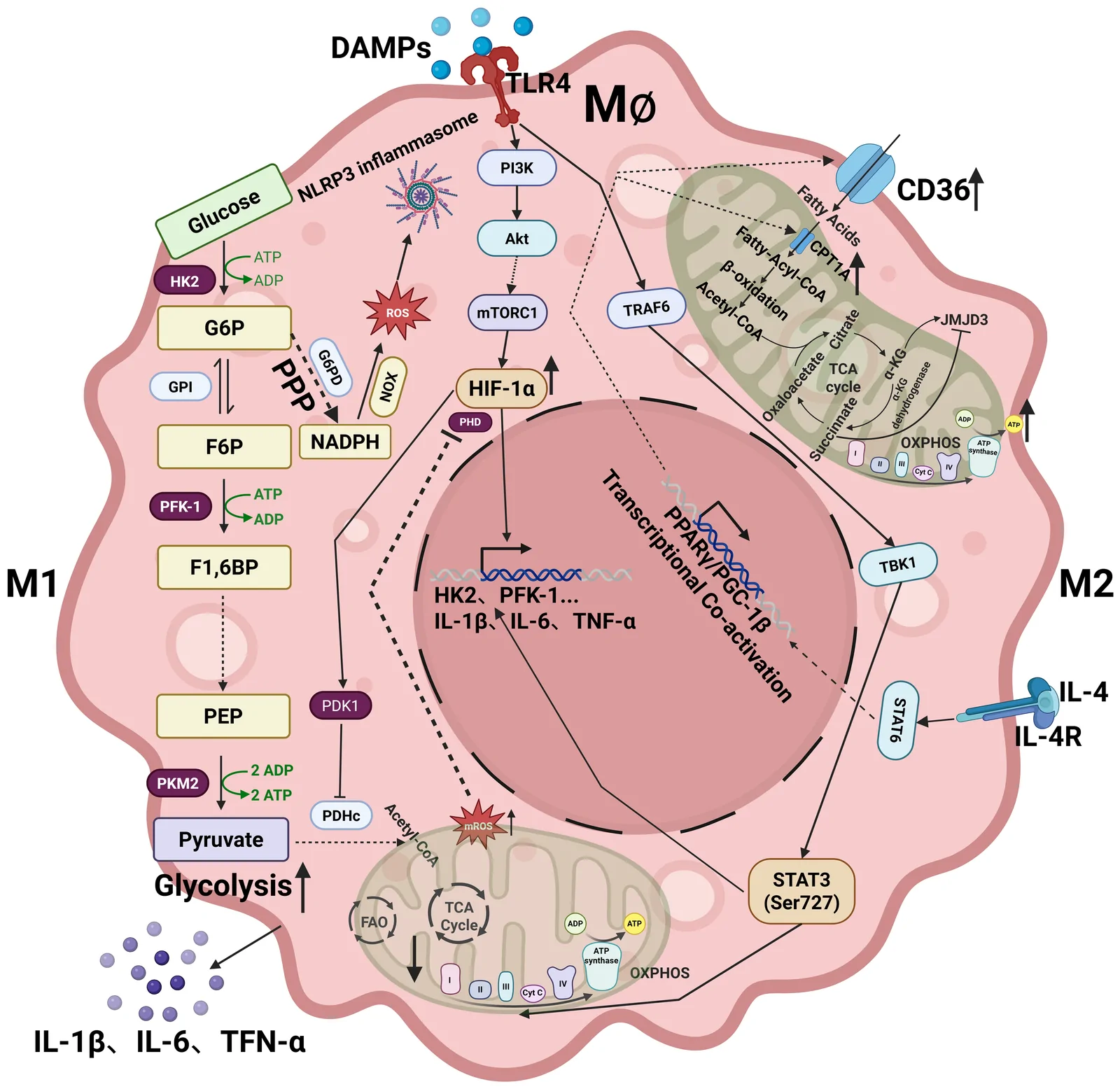
Core mechanisms of metabolic reprogramming in macrophages. Upstream signaling pathways (e.g. PI3K/AKT/mTOR and TRAF6/TBK1/STAT3) promote the transcription and generation of core metabolic enzymes of glycolysis (e.g. HK2, PFK-1) through the activation of transcription factors (e.g. HIF-1α, STAT3), which collectively drive the process of glycolysis and provide fast energy (ATP) and metabolic intermediates for cells. The product of glycolysis, G6P, is catalyzed by G6PD to generate NADPH (PPP pathway), which mediates the generation of reactive oxygen species (ROS) through the enzyme NADPHase (NOX) and activates the NLRP3 inflammatory vesicles, which is the rate-limiting enzyme of PPP. Pyruvate, the end product of glycolysis, is catalyzed by PDHc to generate Acetyl-CoA, which enters the tricarboxylic acid cycle. HIF-1α induces PDK1 to inhibit the entry of pyruvate into the mitochondrial tricarboxylic acid (TCA) cycle, which inhibits the mitochondrial oxidative phosphorylation (OXPHOS) and promotes glycolysis. Stimulation by IL-4 and other signals promotes fatty acid uptake (via upregulation of CD36) and fatty acid oxidation (FAO) through STAT3 signaling, thereby enhancing OXPHOS and driving macrophage polarization toward the anti-inflammatory/repair-oriented M2 phenotype, while concurrently suppressing the production of pro-inflammatory cytokines. The figure also illustrates the metabolic flux among glycolysis, the TCA cycle, FAO, and OXPHOS, as well as their regulatory relationships with macrophage polarization and inflammatory function. Mø, Macrophages; HK2, Hexokinase 2;F6P, Fructose-6-phosphate; PFK-1, Phosphofructokinase-1; F1,6BP, Fructose-1,6-bisphosphate; PEP, Phosphoenolpyruvate; PKM2, Pyruvate kinase M2; PDK-1, Pyruvate Dehydrogenase Kinase 1; PDHc, Pyruvate Dehydrogenase Complex; HIF-1α, Hypoxia-Inducible Factor 1 α; TCA, tricarboxylic acid cycle; FAO, Fatty Acid Oxidation; OXPHOS, Oxidative Phosphorylation; G6PD, Glucose-6-Phosphate Dehydrogenas; PPP, Pentose Phosphate Pathway; NOX, NADPH Oxidase; PI3K, Phosphoinositide 3-Kinase; AKT, Protein kinase B; ROS, Reactive Oxygen Species; mROS, mitochondrial Reactive Oxygen Species; mTORC1, Mammalian Target of Rapamycin Complex 1; TLR4, Toll-Like Receptor 4; TRAF6, TNF Receptor-Associated Factor 6D; TBK1, TANK-Binding Kinase 1; STAT3, Signal Transducer and Activator of Transcription 3; IL-1β, Interleukin-1β; IL-6, Interleukin-6; TNF-α, Tumor Necrosis Factor-α. IL-4, Interleukin-1; IL-4R, Interleukin-4 Receptor; CD36, Cluster of Differentiation 36; STAT6, Signal Transducer and Activator of Transcription 6, PPARγ, Peroxisome Proliferator-Activated Receptor Gamma; CPT1A, Carnitine Palmitoyltransferase 1A, α-KG, Alpha-Ketoglutarate; ATP, Adenosine Triphosphate; ADP, Adenosine Diphosphate; JMJD3, Jumonji Domain-Containing Protein D3.

During acute allograft rejection, pro-inflammatory M1 macrophages predominantly rely on glycolysis to support the production of cytokines such as IL-1β, and TNF-α, thereby amplifying local inflammation and tissue injury (Chen et al., 2024). Conversely, during chronic rejection, M2 macrophages preferentially utilize oxidative phosphorylation (OXPHOS) to sustain the secretion of transforming growth factor beta (TGF-β), promoting fibrosis and vascular remodeling-pathological features that distinguish chronic rejection from acute inflammatory injury (Ochando et al., 2020). Collectively, these findings highlight macrophage metabolic reprogramming as a central mechanism governing cellular phenotype and plasticity. The distinct metabolic characteristics between M1 and M2 macrophages within the graft microenvironment are illustrated in **Figure 1**.

In this review, we systematically summarize the characteristics and functional implications of macrophage metabolic reprogramming in organ transplantation, evaluate its therapeutic potential, and discuss current challenges and future directions.

## 2 Key mechanisms of metabolic reprogramming in macrophages

Metabolic reprogramming is a central driver of macrophage plasticity, with the balance between glycolysis and OXPHOS playing a key role in regulating transitions between pro-inflammatory and anti-inflammatory phenotypes (El Kasmi and Stenmark, 2015). Glycolysis converts glucose to pyruvate through a series of cytosolic enzymatic reactions, generating intermediates that fuel the tricarboxylic acid (TCA) cycle and support biosynthetic processes critical for immune activation. OXPHOS, in contrast, is an oxygen-dependent mitochondrial process that uses nicotinamide adenine dinucleotide (NADH) and flavin adenine dinucleotide (FADH_2_) to generate adenosine triphosphate (ATP) via the electron transport chain and ATP synthase (Viola et al., 2019). Although both pathways form the backbone of cellular energy metabolism, their relative dominance is dynamically regulated by macrophage activation state and by prevailing microenvironmental cues (Kong et al., 2025). Upon stimulation with lipopolysaccharides (LPS) or Interferon-γ (IFN-γ), M1 macrophages preferentially rely on aerobic glycolysis rather than mitochondrial OXPHOS to generate ATP, even under normoxic conditions-a metabolic feature known as the *Warburg* effect. Macrophage metabolism, however, does not operate as a simple binary switch. Macrophages are highly flexible metabolically, and even M1-polarized cells can retain some OXPHOS capacity, the extent of which depends on the intensity of the activating stimulus and the surrounding tissue microenvironment (Geiß et al., 2022; Viola et al., 2019). This metabolic plasticity allows macrophages to adapt rapidly to fluctuating oxygen and nutrient availability within the inflammatory microenvironment (Kolliniati et al., 2022) and sustains the elevated energy and biosynthesis demands of rapid cytokine production, such as IL-1β and TNF-α. This process is tightly regulated at three interdependent levels, metabolic enzymes, transcription factors, and upstream signaling pathways (Lauterbach et al., 2019; Marrocco and Ortiz, 2022).

### 2.1 Metabolic flux and intermediate metabolites as effector and signaling molecules

Several rate-limiting enzymes dictate glycolytic flux and thereby shape macrophage polarization. Hexokinase 2 (HK2), the rate-limiting enzyme that initiates glycolysis, is upregulated in LPS-stimulated macrophages, where it facilitates glucose uptake and conversion to glucose-6-phosphate, provides ATP and metabolic precursors for the synthesis of pro-inflammatory factors (e.g., IL-1β, TNF-α) (Li et al., 2025a; Yu et al., 2022). Phosphofructokinase-1 (PFK-1) is the second rate-limiting enzyme of glycolysis, catalyzes the conversion of fructose 6-phosphate to fructose 1,6-bisphosphate. Its activity is regulated by 6-phosphofructo-2-kinase (PFKFB3), which maintains the intracellular concentration of fructose-2,6-bisphosphate (F-2,6-BP) to allosterically activate PFK -1 and thereby accelerate glycolytic flux (Houddane et al., 2017). Increased expression of PFKFB3 in LPS-stimulated M1 macrophages contributes to increased IL-1β and IL-6 production (Finucane et al., 2019). Pyruvate kinase M2 (PKM2), the third rate-limiting glycolytic enzyme, catalyzes the final step of glycolysis-the conversion of phosphoenolpyruvate to pyruvate. Under inflammatory conditions (e.g., exposure to allograft-derived DAMPs or IFN-γ), PKM2 translocates to the nucleus as a dimer or monomer, forms a complex with HIF-1α, and binds directly to IL-1β promoter to drive its transcription (Palsson-McDermott et al., 2015). In a murine heart transplant rejection model, macrophage-specific mitogen-activated protein kinase kinase 1/2 (MEK1/2)-PKM2 signaling drives the proinflammatory phenotype and aerobic glycolysis of allograft-infiltrating macrophages, whereas MEK1/2 inhibition or genetic ablation significantly reduces inflammatory cytokine production and ameliorates chronic cardiac allograft rejection (Chen et al., 2024). In a mouse model of nonalcoholic fatty liver disease donor liver transplantation, Sirtuin 3 reduced the lactate level in liver macrophages by deacetylating the K367 site of PKM2, thereby attenuating liver fibrosis (Liu et al., 2025). Lactate dehydrogenase (LDHA) catalyzes the conversion of pyruvate to lactate, sustaining glycolytic output and supplying the energy and biosynthetic substrates required for inflammatory responses, and elevated lactate levels, in turn, promote infiltration of proinflammatory macrophages (Kaushik et al., 2019). PDK1, a key regulatory enzyme in glucose metabolism, phosphorylates the components of the mitochondrial pyruvate dehydrogenase complex, thereby suppressing the conversion of pyruvate to acetyl coenzyme A (CoA) and limiting OXPHOS-based energy metabolism (Kaplon et al., 2013; Roche and Hiromasa, 2007).

The TCA cycle is fueled by acetyl-CoA derived from the oxidative decarboxylation of pyruvate and from fatty acid β-oxidation. Acetyl-CoA condenses with oxaloacetate to form citrate, which then undergoes successive decarboxylation and dehydrogenation reactions to generate the intermediates, α-ketoglutarate, succinyl-CoA, succinate, fumarate, and malate, ultimately regenerating oxaloacetate. These reactions produce NADH and FADH2, which transfer electrons to the mitochondrial electron transport chain (ETC) to support OXPHOS and efficient ATP synthesis (Martínez-Reyes and Chandel, 2020). During M1 activation, TCA cycle intermediates are disrupted at specific nodes. IRI-induced hypoxia and DAMP-mediated TLR signaling inhibit succinate dehydrogenase (SDH) via ROS-dependent mechanisms, resulting in succinate accumulation; while reduced isocitrate dehydrogenase (IDH) activity and enhanced citrate export lead to intracellular citrate buildup (Martin et al., 2019; Williams and O'Neill, 2018). In the cytosol of inflammatory macrophages, upregulated ATP-citrate lyase cleaves citrate to produce acetyl-CoA and oxaloacetate, supplying acetyl-CoA for prostaglandin E2 (PGE_2_) synthesis and NADPH (derived from oxaloacetate) for nitric oxide (NO) and ROS production, thereby amplifying the inflammatory response (Infantino et al., 2013). Simultaneously, SDH inhibition allows succinate to accumulate, stabilizing HIF-1α by competitively inhibiting prolyl hydroxylase (PHD) activity and sustaining the pro-inflammatory glycolytic program (discussed further in Section 3.1).

The pentose phosphate pathway (PPP) in macrophages exhibits marked functional heterogeneity. PPP-derived NADPH plays a dual role in redox homeostasis, it maintains glutathione in its reduced state to support antioxidant defense, while also driving ROS generation via NADPH oxidase (NOX) to sustain pro-inflammatory responses. Early allorecognition-induced cytokines (e.g., IFN-γ) and ROS upregulate the rate-limiting enzyme Glucose-6-phosphate dehydrogenase (G6PD) via NF-κB signaling, shunting glucose-6-phosphate into the PPP to generate NADPH for NOX-dependent respiratory bursts and antioxidant defense, thereby supporting NO production and promoting NLRP3 inflammasome activation (Galván-Peña and O'Neill, 2014; Ghergurovich et al., 2020). G6PD is negatively regulated by carbohydrate kinase-like protein (CARKL); LPS-mediated inhibition of CARKL relieves this repression of PPP and thereby promotes M1 polarization (Ham et al., 2016; Park et al., 2017).

FAO is the metabolic hallmark of M2 macrophages. In the chronic rejection or fibrotic microenvironment, cytokines such as IL-4 activate the Signal Transducer and Activator of Transcription 6 (STAT6)/peroxisome proliferator-activated receptor γ (PPARγ) axis, upregulating carnitine palmitoyltransferase 1A (CPT1A) to drive FAO as fuel for OXPHOS and thereby direct the phenotypic shift toward M2-like reparative macrophages (Nomura et al., 2016; Vassiliou and Farias-Pereira, 2023). PPARγ, together with PGC-1β, synergistically activates FAO-related genes, including the fatty acid translocase CD36, to enhance lipid uptake and catabolism (Liu et al., 2021). mTORC2-mediated AKT signaling promotes lipid droplet synthesis, and pharmacological or genetic inhibition of FAO suppresses M2 polarization, whereas exogenous fatty acid supplementation promotes M2 activation (Huang et al., 2016). The role of FAO, however, extends beyond the M2 phenotype. Under chronic lipid overload (lipotoxicity), elevated saturated fatty acids such as palmitate overwhelm mitochondrial oxidative capacity, leading to incomplete FAO and accumulation of toxic lipid intermediates (e.g., ceramides, acylcarnitines) that activate pro-inflammatory signaling cascades including c-Jun N-terminal kinase (JNK) and NF-κB (Kruglov et al., 2024; Prieur et al., 2010). In aging and metabolically stressed microenvironments, M1-like macrophages exhibit heightened FAO activity as an adaptive response to detoxify excess lipids, wherein enhanced FAO supports sustained pro-inflammatory cytokine production (TNF-α, IL-6, IL-1β) through NLRP3 inflammasome activation (Kruglov et al., 2024). Thus, FAO displays functional plasticity: it drives the anti-inflammatory M2 program under homeostatic cytokine cues, yet fuels pro-inflammatory macrophage activation under lipotoxic stress. Notably, pharmacological targeting of FAO has been evaluated in solid organ transplantation settings. Inhibition of fatty acid β-oxidation with etomoxir (a CPT1A inhibitor) impairs pro-inflammatory monocyte differentiation and markedly prolongs heart allograft survival in murine models (Zhu et al., 2022). Collectively, these findings establish FAO as a central, context-dependent metabolic determinant of macrophage phenotypic plasticity, with emerging pharmacological interventions demonstrating translational potential in transplantation immunology.

Glutamine catabolism produces α-ketoglutarate (αKG) which is important for M2 activation in macrophages. α-KG serves as an essential co-factor for histone demethylase (Jumonji domain-containing protein-3, Jmjd3), which catalyzes the demethylation of H3K27me3 (repressive histone mark), thereby relieving transcriptional repression of M2 marker genes (e.g., Arg1, Ym1) and promoting an anti-inflammatory phenotype (Liu et al., 2017). Arginine metabolism also differs between M1 and M2 macrophages, M1 macrophages express inducible nitric oxide synthase (iNOS), which metabolizes arginine to NO and citrulline for antimicrobial functions, whereas M2 macrophages express arginase-1 (ARG-1), which metabolizes arginine to ornithine and urea to supply substrates for cell proliferation and tissue repair.

### 2.2 Transcription factors and signaling nodes orchestrating metabolic reprogramming

Transcription factors coordinate the expression of glycolytic enzymes in response to inflammatory signals. In implanted organs, IRI creates profound tissue hypoxia, and pro-inflammatory stimuli such as LPS from gut bacterial translocation stabilize HIF-1α even under normoxic conditions (Serra-Pérez et al., 2010). HIF-1α upregulates glycolytic genes including HK2 and PFKFB3, while also inducing PDK1 to suppress the TCA cycle, thereby promoting glycolysis and limiting OXPHOS (Wang et al., 2026, 2017). In addition, HIF-1α influences epigenetic remodeling, elevated glycolysis and accumulation of TCA intermediates (e.g., fumarate, succinate) enhance histone methylation (H3K4me3) and acetylation (H3K27ac), contributing to the phenomenon of “trained immunity”, a phenomenon characterized by enhanced cytokine production upon secondary stimulation (Ochando et al., 2023; Rius et al., 2008). While IκB kinase-β (IKK-β) promotes HIF-1α transcription to maintain glycolysis by activating NF-κB, the NF-κB subunit RelA supports OXPHOS under specific circumstances, highlighting the subtle cross-talk between signaling and metabolism (Li et al., 2024; Rius et al., 2008). TLR4 induces STAT3 Ser727 phosphorylation through TNF receptor-associated factor 6 (TRAF6)/TANK-binding kinase 1 (TBK1) signaling axis. This non-canonical activation drives inflammatory macrophages from OXPHOS to aerobic glycolysis, accompanied by succinate accumulation (Balic et al., 2020).

Signaling pathways regulate the expression of core metabolic enzymes and transcription factors by integrating internal and external cues, thereby synergistically promoting glycolysis-driven metabolic reprogramming and macrophage polarization. In response to allograft-derived signals, such as growth factors and amino acids from necrotic debris, mTORC1 promotes HIF-1α translation and activates glycolytic gene expression, including *Glut1*, *Pfkp*, and *Pdk1*, by phosphorylating eukaryotic translation initiation factor 4E-binding protein 1 (4E-BP1), thereby driving glycolytic metabolism (Düvel et al., 2010). Akt increases cell-surface expression of GLUT1 and promotes hexokinase phosphorylation, substantially increasing glycolytic flux in macrophages (Robey and Hay, 2009).

The metabolic profile of M2-type macrophages is centered on OXPHOS and relies on FAO and the PPP for synergistic coordinated supply of energy and biosynthetic precursors. Key factors such as PPARγ and adenosine 5’-monophosphate (AMP)-activated protein kinase (AMPK) support anti-inflammatory and tissue repair functions by regulating metabolic enzyme activity and gene expression. PPARγ promotes fatty acid uptake and β-oxidation (discussed in Section 2.1), maintaining the energetic homeostasis required for M2 polarization (El Kharbili et al., 2022). AMPK directly phosphorylates PGC-1α, promoting its nuclear translocation and enhancing mitochondrial DNA replication and respiratory chain complex expression, thereby boosting OXPHOS capacity; conversely, mTORC1 phosphorylates Unc-51 Like Autophagy Activating Kinase 1 (ULK1), the autophagy-initiating kinase, blocking the formation of autophagic vesicles, which leads to accumulation of dysfunctional mitochondria and reduced OXPHOS efficiency (Pagán et al., 2022; Sun et al., 2024).

In summary, macrophage metabolic reprogramming dynamically regulates polarization state through the synergistic or antagonistic interplay of glycolysis, OXPHOS, the TCA cycle, PPP, FAO and amino acid metabolism. Key metabolic enzymes (e.g., HIF-1α, PKM2, PPARγ) and metabolites (e.g., succinate, α-KG, lactate) achieve functional plasticity through signaling pathways (STAT, mTOR, AMPK) and epigenetic mechanisms (histone modifications).

## 3 Role of macrophage metabolism in organ transplantation

**Figure 2:**
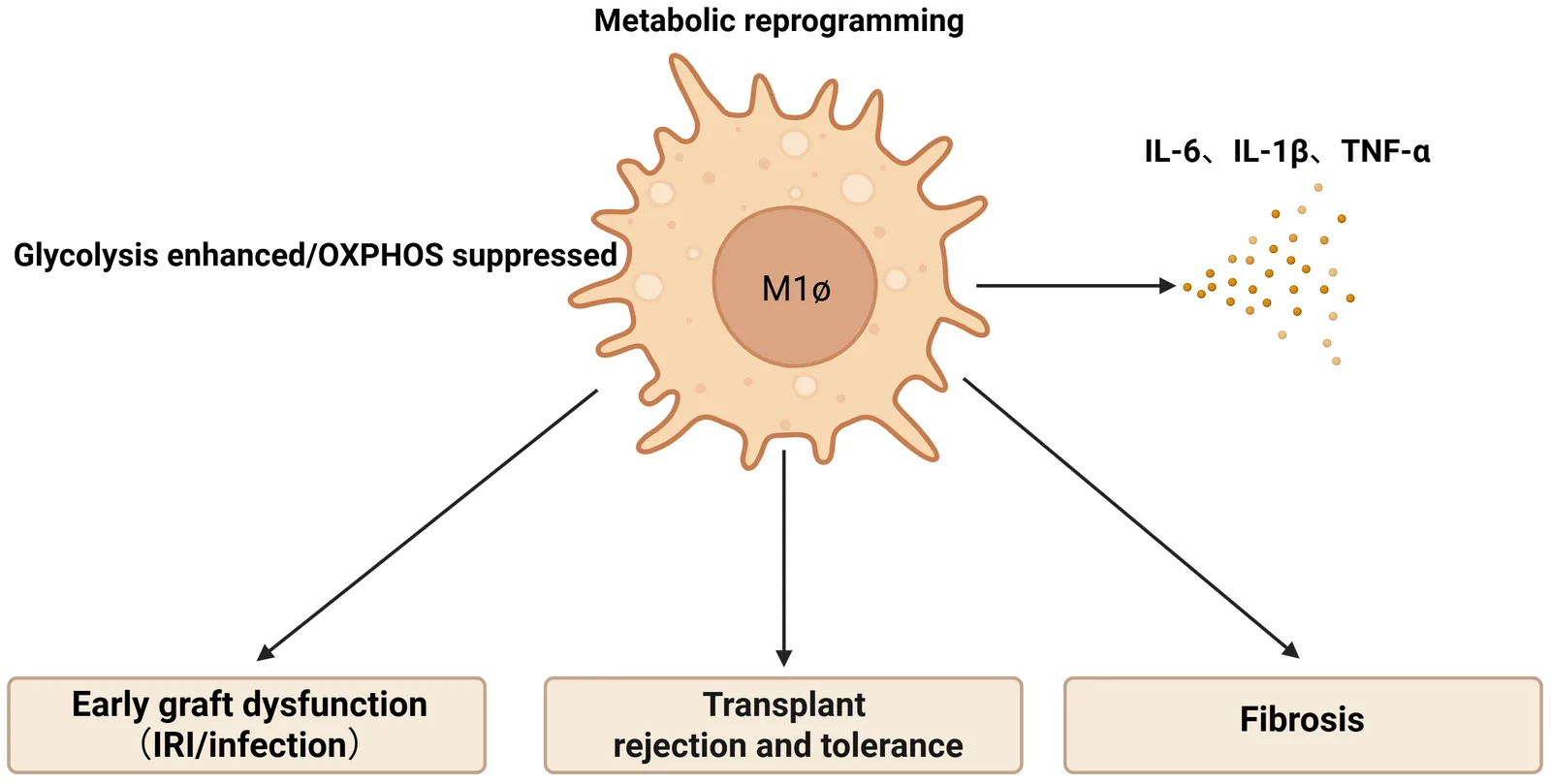
Role of M1 macrophage metabolic reprogramming in organ transplantation. M1 macrophages exhibit enhanced glycolysis and suppressed oxidative phosphorylation through metabolic reprogramming, and this metabolic state promotes the release of pro-inflammatory cytokines IL-6, IL-1β, and TNF-α by M1 macrophages, which are involved in early graft dysfunction (ischemia-reperfusion injury or infection), graft rejection and immune tolerance imbalance, and tissue fibrosis. IRI, ischemia-reperfusion injury; IL-1β, Interleukin-1β; IL-6, Interleukin-6; TNF-α, Tumor Necrosis Factor-α.

Macrophage metabolic reprogramming in transplantation is triggered by upstream signals-including alloantigen recognition, DAMPs, and pro-inflammatory cytokines (e.g., IL-1β)-via cascades such as TLR4/NF-κB and PI3K/Akt/mTOR. Rather than initiating alloimmunity, metabolic reprogramming serves as a downstream effector that amplifies inflammation. Once established, however, it generates positive feedback (e.g., lactate and succinate stabilize HIF-1α) that sustains graft inflammation beyond the initial trigger. Recognizing this hierarchical relationship has clinical value, as it uncovers metabolic nodes (e.g., PKM2, CPT1A) that can be therapeutically modulated to attenuate inflammation and prolong graft survival without necessarily disrupting upstream alloimmune recognition. The role of metabolic reprogramming of M1 macrophages in organ transplantation is illustrated in **Figure 2**.

### 3.1 Impact of macrophage metabolic reprogramming on early graft dysfunction

Early graft dysfunction is precipitated by multiple insults, of which IRI and perioperative infection are the primary contributors. During IRI, macrophages undergo distinct metabolic adaptations depending on the phase and severity of ischemic injury. The hypoxic microenvironment of both cold and warm ischemia, together with DAMPs released from injured graft tissues, drives macrophage polarization toward the M1 phenotype. This polarization is characterized by markedly enhanced glycolytic flux and activation of the MEK/ERK-PKM2 signaling axis, which amplifies the release of pro-inflammatory cytokines including IL-1β and TNF-α (Chen et al., 2024). Succinate plays a central mechanistic role in IRI, during ischemia, obstruction of the TCA cycle causes substantial succinate accumulation and upon reperfusion, this accrued succinate is rapidly reoxidized by SDH, driving reverse electron transport at mitochondrial complex I and triggering a robust burst of ROS (Li et al., 2025b). The interruption of oxygen supply during ischemia directly suppresses OXPHOS, whereas the reperfusion-induced ROS surge further damages respiratory chain complexes, together causing severe impairment of ATP synthesis. In response to OXPHOS failure, macrophages shift toward glycolytic ATP production. However, the reperfusion-associated ROS burst depletes the already limited pools of NAD^+^ and ATP, rendering even this compensatory glycolysis unsustainable and ultimately leading to glycolytic ATP exhaustion (Chouchani et al., 2014; Leng et al., 2025; Yakupova et al., 2022). As a TCA intermediate, accumulated succinate additionally stabilizes HIF-1α by inhibiting PHD activity (Oh et al., 2023; Tannahill et al., 2013). Under hypoxia, redox perturbations within the mitochondrial ETC promote the reassembly of PKM2 oligomers into dimers that translocate to the nucleus, phosphorylate STAT3, and drive expression of downstream inflammatory mediators including IL-6 and IL-1β (Ao-Di et al., 2024; Guo et al., 2021; Shirai et al., 2016). In M1 macrophages undergoing IRI, a portion of the TCA cycle is redirected through the aspartate-arginine-succinate shunt, coordinating arginine production with NO synthesis. Aspartate aminotransferase, the key enzyme in this shunt, stimulates NO and IL-6 production in inflammatory macrophages while simultaneously inhibiting mitochondrial respiration (Jha et al., 2015). These findings underscore the importance of the early post-reperfusion timepoint as a therapeutic target. Targeting macrophage glycolysis with agents such as dimethyl fumarate, which activates the NRF2 pathway and restores mitochondrial homeostasis, within the first 24 hours post-reperfusion represents a promising intervention window for attenuating IRI (Tang et al., 2022).

During perioperative infection, macrophages exposed to pathogen-associated molecular patterns (PAMPs) additionally undergo epigenetic and metabolic reprogramming consistent with trained immunity, characterized by upregulation of glycolytic enzymes (e.g., HK2, PFKFB3), inhibition of SDH, and accumulation of succinate. Succinate suppresses α-KG-dependent histone demethylases, elevating H3K4me3 at pro-inflammatory gene loci and thereby stabilizing a persistent inflammatory transcriptional state (Arts et al., 2016; Ochando et al., 2020).

In summary, M1 macrophage polarization during early graft dysfunction is mediated by enhanced glycolytic metabolism, mitochondrial ROS bursts, and TCA cycle remodeling, culminating in amplified cytokine release, endothelial activation, and tissue injury.

### 3.2 Impact of macrophage metabolic reprogramming on allograft rejection and tolerance

Macrophage metabolic status critically influences the balance between allograft rejection and immune tolerance, functioning as an amplifier of upstream alloimmune signals. During acute allograft rejection, tissue-resident macrophages are progressively supplanted by glycolysis-dominant M1 macrophages, which release pro-inflammatory cytokines (IL-6, TNF-α) and upregulate endothelial adhesion molecules such as vascular cell adhesion molecule-1 (VCAM-1), promoting T cell infiltration into the graft (Liu et al., 2016). HIF-1α is a key downstream mediator of this response. Pharmacological HIF-1α inhibition attenuates macrophage antigen-presenting capacity and pro-inflammatory cytokine production, and reduces CD4^+^ and CD8^+^ T cell infiltration during acute allograft rejection (Chang et al., 2021). ^18^F-FDG PET imaging could be used to noninvasively detect these metabolic shifts in macrophage-rich infiltrates, serving as an early diagnostic marker for ongoing immune-mediated rejection (Reuter et al., 2010).

Chronic rejection is a slow, progressive process characterized by persistent vascular remodeling, tissue fibrosis, and irreversible loss of graft function. During this process, infiltrating macrophages undergo profound metabolic reprogramming, with their polarization status and functional phenotype dynamically reshaped accordingly (Uosef et al., 2026). As fibrosis advances, macrophages frequently exhibit a mixed M1/M2 metabolic state, reflecting their adaptive response to the continuously evolving graft microenvironment (Chen et al., 2025b). Although macrophage plasticity underlies pathological injury, it also offers a potential therapeutic window for intervention (Uosef et al., 2026). Metabolic crosstalk between T cells and macrophages likewise significantly influences the development and progression of chronic rejection. Lactate produced by activated T cells promotes M2 polarization of macrophages through histone lysine lactylation (Noe et al., 2021). MiR-21 is markedly overexpressed in chronic allograft vasculopathy (CAV) following heart transplantation, and targeted inhibition of miR-21 effectively delays CAV progression by reprogramming macrophage metabolism-promoting OXPHOS, suppressing glycolysis, and increasing the abundance of M2-like macrophages (Usuelli et al., 2021). Furthermore, the MEK1/2-PKM2 axis drives metabolic reprogramming of graft-infiltrating macrophages, enhancing glycolytic metabolism and promoting a pro-inflammatory phenotype that accelerates chronic rejection; whereas blockade of this signaling axis significantly ameliorates chronic cardiac allograft rejection (Chen et al., 2024).

Changes in metabolic pathways also direct macrophage differentiation toward a pro-tolerogenic state, which is critical for both the establishment and maintenance of immune tolerance. HIF-2α transcriptionally activate CSF1R expression, facilitating the differentiation of monocytes into tolerogenic macrophages, and myeloid-specific HIF-2α expression is required for the successful induction of tolerance. Furthermore, administration of a HIF-2α agonist enhances its expression and improves graft survival (DeBerge et al., 2024). In a mouse heart transplantation model, erythropoietin was found to counteract trained immunity, mitigating inflammatory responses and fostering immune tolerance by reversing the metabolic and epigenetic alterations induced by trained immunity in macrophages, thereby prolonging allograft survival (Bin et al., 2026).

### 3.3 Impact of macrophage metabolic reprogramming on long-term graft function

Metabolic reprogramming of macrophages significantly affects long-term graft function at distinct stages along the post-transplantation timelines, including IRI-associated delayed graft function (DGF), chronic rejection leading to interstitial fibrosis, graft vasculopathy and post-transplant tumor recurrence.

In IRI-mediated DGF, macrophages are rapidly activated toward a pro-inflammatory M1 phenotype that relies on aerobic glycolysis for rapid energy production to support fulminant inflammatory responses. This amplifies the release of pro-inflammatory cytokines, including IL-1β and TNF-α, thereby exacerbating tissue injury and promoting DGF development. As inflammation resolves, macrophage metabolic programming shifts toward OXPHOS and FAO to meet the energy and biosynthetic demands of reparative functions (Ao-Di et al., 2024). Growth differentiation factor 15, derived from renal tubular epithelial cells, limits kidney transplant injury by reprogramming macrophage responses-suppressing pro-inflammatory M1 polarization and promoting reparative M2 polarization (Shi et al., 2026). Clinical protocol biopsies obtained within 24 hours post-transplant have shown that macrophage density in early surveillance biopsies predicts future renal transplant function, with higher infiltration of glycolytic macrophages correlating with worse graft outcomes at one year (Bräsen et al., 2017).

Metabolic reprogramming in macrophages contributes to fibrosis in chronic rejection by sustaining the metabolic demands of M2 polarization, facilitating macrophage-to-myofibroblast transition (MMT), and engaging epigenetic mechanisms. Importantly, these metabolic changes are initiated by upstream signals-including TGF-β1 from the fibrotic microenvironment and sustained inflammatory cytokines-and in turn reinforce the pro-fibrotic program through positive feedback. Although M2-type macrophages possess anti-inflammatory and tissue-reparative properties, in chronic rejection, their altered metabolic pathways including OXPHOS and FAO - supply the energetic and biosynthetic substrates that sustain their profibrotic function. These metabolic adaptations enable M2 macrophages to secrete profibrotic factors such as TGF-β1 and platelet-derived growth factor, which directly activate fibroblast proliferation and enhance extracellular matrix deposition, thereby exacerbating graft fibrosis (Uosef et al., 2026). Sustained HIF-1α activity in macrophages promotes a profibrotic transcriptional program by directly binding to hypoxia-responsive elements in target genes, thereby amplifying the expression of TGF-β1 and connective tissue growth factor (Higgins et al., 2007). Transdifferentiation of macrophages, particularly the M2 subtype into myofibroblasts, represents another critical cellular pathway in graft fibrosis. Studies of renal biopsy samples from patients with chronic active renal allograft rejection (CAR) have demonstrated that methyltransferase 3 (METTL3) enhances M2 to MMT via the TGF-β1/Smad3 signaling axis; and that inhibiting METTL3 or its downstream target Smad3 abrogates this process and alleviates graft fibrosis (Yao et al., 2025). In chronic allograft rejection, activation of the TGF-β1/Smad3 pathway drives MMT, promoting collagen deposition and interstitial fibrosis; Smad3 knockout effectively protects renal allografts from interstitial fibrosis and markedly reduces the number of macrophage-derived myofibroblasts (Wang et al., 2017b). Epigenetic modifications also play a pivotal role in the pro-fibrotic program of macrophages. In chronic rejection, histone deacetylases (HDACs) are involved in regulating macrophage polarization and their fibrosis-related functions (Halasa and Wawruszak, 2024). Analysis of single-cell transcriptomic data from human heart allografts revealed that macrophages accumulating in fibrotic grafts undergo reprogramming mediated by histone methylation - specifically, histone methyltransferase Setdb1 regulates pro-fibrotic macrophages reprogramming through H3K9 methylation, and macrophage-specific ablation of Setdb1 suppresses the reprogramming of pro-fibrotic macrophage subsets such as Fn1^+^ and SPP1^+^, thereby mitigating fibrosis and prolonging graft survival (Ma et al., 2025). Furthermore, in human renal allograft biopsy samples collected one-year post-transplant, M2-activated macrophages have been found to constitute up to 92% of all infiltrating macrophages (Toki et al., 2014).

Cardiac and renal allograft vasculopathy represent a distinct manifestation of chronic rejection, characterized by progressive intimal hyperplasia and vascular remodeling driven in part by macrophage-mediated inflammatory and metabolic signaling. In CAV, macrophages within the graft vasculature undergo metabolic reprogramming that sustains chronic inflammation and drives smooth muscle cell proliferation. miR-21, a microRNA robustly upregulated in both murine and human CAV, serves as a key metabolic regulator; antagonism of miR-21 has been shown to reprogram macrophage metabolism by restoring OXPHOS and inhibiting aberrant glycolysis, which in turn attenuates CAV progression and delays its onset (Usuelli et al., 2021). Recent studies have revealed that CD8^+^ T cell-derived soluble factors induce cleavage of the macrophage efferocytosis receptor MerTK, which subsequently rewires macrophage metabolism toward a pro-inflammatory glycolytic state, accelerating cardiac allograft vasculopathy (Shah et al., 2026). In human cardiac allograft biopsies, the degree of macrophage infiltration and the expression of glycolytic enzymes within graft-infiltrating leukocytes correlate with the severity of intimal thickening and clinical CAV progression (Usuelli et al., 2021).

Unlike active rejection, chronic graft dysfunction encompasses interstitial fibrosis/tubular atrophy (IF/TA) and transplant vasculopathy, which may occur in the absence of a sustained alloimmune attack (Haas, 2014). Persistent FAO impairment in M2-like macrophages contributes to defective efferocytosis and impaired matrix remodeling, allowing apoptotic debris to accumulate and sustain the fibrotic response (Granata et al., 2020). The efferocytic capacity of M2 macrophages depends on FAO-derived energy; when FAO is impaired, macrophages fail to clear apoptotic cells efficiently, further perpetuating inflammation and tissue injury. Mitochondrial dysfunction represents an additional driver of chronic injury (Zhang et al., 2019). Drp1-mediated mitochondrial fission promotes macrophage metabolic reprogramming toward a pro-inflammatory state, impairing the resolution phase of tissue repair and contributing to chronic graft damage (Yadav et al., 2025). Dysregulated mitochondrial dynamics-characterized by excessive fission and impaired fusion-compromise the metabolic plasticity of macrophages, preventing their transition from pro-inflammatory to pro-repair phenotypes (He and Sun, 2026).

With respect to post-transplant tumor recurrence, macrophage metabolic reprogramming significantly reshapes the immunosuppressive microenvironment. During the late post-transplant period, long-term immunosuppressive therapy (e.g., tacrolimus and rapamycin), along with the subsidence of chronic inflammation, facilitates the dominance of M2-type macrophages, which, supported by FAO and OXPHOS, generate immunosuppressive mediators (IL-10, TGF-β) and express PD-L1, thereby suppressing T cell function. Concurrent VEGF secretion by M2 macrophages promotes neovascularization, collectively enhancing the risk of post-transplant malignancy (Chu et al., 2024; Jin et al., 2025).

## 4 Potential therapeutic strategies targeting macrophage metabolism

### 4.1 Small-molecule metabolic inhibitors

Macrophage-specific metabolic alterations are primary drivers of M1 effector function, making inhibition of key glycolytic enzymes an attractive therapeutic approach to blunt pro-inflammatory polarization and attenuate post-transplant inflammatory responses. 2-deoxyglucose (2-DG), a structural glucose analogue, competitively inhibits hexokinase. In the mouse lung transplantation model, 2-DG can significantly prevent chronic lung allograft dysfunction by inhibiting macrophage glycolysis (Cano et al., 2025). Etomoxir, an irreversible inhibitor of CPT1A, blocks FAO, which impairs monocyte-to-dendritic cell and monocyte-to-macrophage differentiation, reduces early T cell infiltration and activation, and prolongs heart allograft survival in a manner dependent on monocyte mobilization, as confirmed by Ccr2-deficient recipients and conditional CPT1A deletion (Zhu et al., 2022). Yeboah et al. used a humanized mouse allogeneic transplantation model to induce tolerance *in vivo* by reprogramming monocytes to an M2-like immunosuppressive phenotype with significant up-regulation of genes involved in OXPHOS by intervention with a LILRB3 agonist antibody (clone A1) (Yeboah et al., 2020). However, FAO is also an important metabolic pathway of regulatory T (Treg) cells, which are highly dependent on FAO-driven OXPHOS for their suppressive function and “effector” differentiation (McDonald-Hyman et al., 2025). Therefore, targeting FAO alone in the treatment of transplant rejection may have the potential risk of weakening Treg-mediated immune tolerance and aggravating graft rejection. Trametinib, a selective MEK1/2 inhibitor, ameliorates chronic heart transplant rejection in a mouse model by blocking PKM2 nuclear translocation (Chen et al., 2024).

### 4.2 Cell therapies

Graft-resident macrophage-derived extracellular vesicles (EVs) represent a powerful mechanism of intercellular communication in the allograft microenvironment. CD63^+^ graft-resident macrophage-derived EVs carry miR-363 and miR-709. miR-363 promotes M1 polarization in the recipient spleen by targeting the 3′ UTR of Fcho2 mRNA, activating the Fcho2/Notch1 signaling axis and thereby amplifying acute allograft rejection (Zheng et al., 2025). These findings illuminate EV-mediated intercellular metabolic communication as a potential target for mitigating transplant rejection. Mesenchymal stem cells-derived exosomes carrying soluble Fgl2 activate the SHP2/STAT3 signaling axis via the CD32b receptor, thereby reprogramming macrophages toward an M2 anti-inflammatory phenotype and effectively mitigating acute rejection in a murine cardiac transplantation model (Chen et al., 2025a).

### 4.3 Nanoparticle or gene-editing platforms

Despite improvements in managing cardiac transplantation, CAV remains a major obstacle to long-term graft survival. Usuelli et al. demonstrated that antagonism of miR-21 inhibits macrophage glycolysis through an arginase-dependent pathway, induces a switch from an M1-like to an M2 metabolic profile, and delays CAV progression in murine models (Usuelli et al., 2021). Braza et al. developed an HDL nanoparticle-based delivery system for precision targeting of the mTOR metabolic pathway and the CD40-TRAF6 co-stimulatory axis in macrophages, selectively blocking aerobic glycolysis and pro-inflammatory cytokine production. The resulting regulatory macrophages inhibited alloreactive CD8^+^ T cell-mediated immune attack and promoted expansion of tolerogenic CD4^+^ Tregs, achieving long-term immune tolerance in experimental transplant models (Braza et al., 2018). Macrophage-specific Mek1/2 knockout mice generated by CRISPR/Cas9 gene editing exhibited markedly reduced pro-inflammatory phenotype and glycolytic capacity upon LPS and IFN-γ stimulation and were significantly protected against chronic cardiac allograft rejection (Chen et al., 2024). Li et al. used CRISPR/Cas9 gene editing to knock out TDO2 gene in rat liver transplantation model, altering the tryptophan-kynurenine metabolism pathway (resulting in reduced kynurenine production) of macrophages through the TDO2-kynurenine (Kyn)-aryl hydrocarbon receptor signaling pathway, this promotes polarization toward proinflammatory M1 macrophages, with concomitant depletion of M2-like macrophages, which in turn exacerbates acute graft rejection and substantially reduces graft survivals (Li et al., 2025c). Myeloid-specific knockout of the epigenetic regulator Setdb1 in macrophages significantly reduced graft fibrosis and prolonged graft survival by blocking the epigenetic reprogramming of Fn1^+^ and SPP1^+^ profibrotic macrophages driven by CCR2-Creb/Setdb1 axis in a mouse heart transplantation model (Ma et al., 2025).

The aforementioned preclinical interventions-ranging from small-molecule inhibitors and biological agents to nanoparticle delivery and gene editing-are summarized and compared in **Table 1**, which highlights their distinct metabolic targets, experimental models, and impacts on graft survival.

**Table 1:** Therapeutic strategies in preclinical animal models targeting macrophage metabolism.

Type	Medication/modality of intervention	Animal models	Effect of metabolic process	Effect on graft survival	Ref
Small-molecule metabolic inhibitors	2-Deoxyglucose (N/A)	Mouse orthotopic lung transplant model	Inhibits glycolysis (competitively inhibits hexokinase)	Significantly prevents chronic lung allograft dysfunction	(Cano et al., 2025)
	Etomoxir (i.p.)	Mouse heart transplantation	Inhibits fatty acid β-oxidation (inhibits carnitine palmitoyltransferase 1)	Prolongs median graft survival from 8 to 13 days	(Zhu et al., 2022)
	LILRB3 Agonist Antibody (Clone A1) (i.v.+i.p.)	humanized mouse allogeneic transplantation model	Enhances OXPHOS (activates inhibitory receptor LILRB3)	The allogeneic lymphoma cells survived, engrafted, and grew successfully	(Yeboah et al., 2020, 2020)
	Trametinib (i.g.)	Mouse heart transplantation	Shifts glycolysis toward OXPHOS (inhibits MEK1/2)	Prolongs median graft survival from 55 to 85 days	(Chen et al., 2024)
Biological agents	Erythropoietin (i.p.)	Mouse heart transplantation	Reverses trained immunity-induced metabolic and epigenetic alterations; restores OXPHOS	Prolongs median graft survival from 35 to more than 100 days	(Bin et al., 2026)
Cell-derived biologics	Mesenchymal stem cell derived Exosomes (sFgl2-MSC-Exos)	Mouse heart transplantation	Reprograms M1 to M2 polarization (binds to CD32b receptor on macrophages)	Prolongs median graft survival from 7 to 23 days	(Chen et al., 2025a)
Nanoparticle or gene-editing platforms	High-density lipoprotein (HDL) nanocarrier targeted delivery systems	Mouse heart transplant model	Selectively inhibits mTOR signaling in macrophages	Prolongs median graft survival from 7 to more than 100 days	(Braza et al., 2018)
	miR-21 conditional deletion	Mouse model of chronic cardiac allograft vasculopathy	Shifts glycolysis toward OXPHOS	Prolongs median graft survival from 60 to more than 95 days	(Usuelli et al., 2021)
	Macrophage-specific Mek1/2 knockout mice	Mouse heart transplant model	Inhibits glycolysis	Prolongs median graft survival from 55 to more than 100 days (In a model of chronic rejection)	(Chen et al., 2024)
	Myeloid-specific Setdb1 knockout	Mouse heart transplant model	Blocks epigenetic reprogramming of profibrotic macrophages	Prolongs median graft survival from 80 to more than 150 days	(Ma et al., 2025)

## 5 Challenges and future directions

Macrophage metabolic reprogramming represents a conceptually powerful framework for improving transplantation outcomes, yet several critical challenges must be addressed before clinical translation becomes feasible.

A major challenge in attributing transplantation outcomes solely to macrophage metabolism lies in the metabolic interdependence of multiple leukocyte and parenchymal cell subsets. For example, glycolytic reprogramming in T cells and endothelial cells also contributes to IRI and rejection, and the metabolic byproducts of one cell type can serve as signaling molecules for another. Therefore, the metabolic changes observed in bulk allograft tissue may not reflect macrophage-intrinsic alterations. Whenever possible, we have prioritized studies employing macrophage-specific gene editing, cell depletion, or targeted nanodelivery to establish causality; However, such studies remain limited. Future investigations should integrate lineage tracing with single-cell metabolic flux analysis to dissect cell-autonomous versus community-level metabolic effects.

Target specificity is a primary concern. Conventional metabolic drugs-such as the glycolytic inhibitor PFK-158 or mTOR inhibitors-may non-selectively impair the function of other immune cell populations, including T cells and dendritic cells, potentially disrupting the homeostatic balance of immunosuppression required for graft acceptance. Precision delivery platforms such as mTOR inhibitor-loaded HDL nanoparticles (mTORi-HDL) offer a promising solution by achieving macrophage-selective metabolic modulation without broadly perturbing T cell metabolic pathways (Braza et al., 2018). Gene editing approaches, including CRISPR/Cas9-mediated targeting of metabolic nodes such as the MEK/ERK-PKM2 axis, provide an additional layer of cell-type specificity (Chen et al., 2024).

Phenotypic complexity presents another major challenge. Macrophage polarization along the M1/M2 spectrum is highly context-dependent and shaped by dynamic microenvironmental metabolic signals-including succinate, α-KG, and lactate-and the coexistence of intermediate phenotypic states (e.g., M2a, M2b, M2c) with overlapping metabolic signatures substantially increases the complexity of therapeutic targeting. A more refined, continuum-based understanding of macrophage metabolic states-informed by single-cell transcriptomics, spatial metabolomics, and multiparametric flow cytometry-will be essential.

Translational biomarkers remain underdeveloped. Macrophage metabolic signatures-including glycolytic enzyme PKM2 and the pro-inflammatory mediator iNOS, hold promise as early diagnostic markers of allograft rejection, but standardized detection platforms and clinically validated thresholds are still lacking. Integration of single-cell spatial metabolomics with artificial intelligence-driven predictive models offers the prospect of resolving the local metabolic microenvironment of transplanted organs with high resolution, revealing macrophage-parenchymal cell metabolic interaction networks, and enabling dynamic prediction of metabolic pathway trajectories during the post-transplant course.

Looking ahead, the field must move toward integration of multi-omic datasets, encompassing the metabolome, epigenome, transcriptome, and proteome, to construct precision treatment algorithms that account for individual metabolic heterogeneity. Ultimately, realizing the therapeutic potential of macrophage metabolic reprogramming to confer durable transplantation immune tolerance will require collaborative efforts spanning fundamental immunology, metabolic biology, nanotechnology, gene editing, and clinical transplantation medicine.
